# The effects of neighborhood density and street connectivity on walking behavior: the Twin Cities walking study

**DOI:** 10.1186/1742-5573-4-16

**Published:** 2007-12-13

**Authors:** J Michael Oakes, Ann Forsyth, Kathryn H Schmitz

**Affiliations:** 1 Division of Epidemiology & Community Health, University of Minnesota, Minneapolis, MN, USA; 2 Department of City and Regional Planning, Cornell University, Ithaca, NY, USA; 3 Division of Epidemiology, University of Pennsylvania School of Medicine, Philadelphia, PA, USA

## Abstract

A growing body of health and policy research suggests residential neighborhood density and street connectivity affect walking and total physical activity, both of which are important risk factors for obesity and related chronic diseases. The authors report results from their methodologically novel Twin Cities Walking Study; a multilevel study which examined the relationship between built environments, walking behavior and total physical activity. In order to maximize neighborhood-level variation while maintaining the exchangeability of resident-subjects, investigators sampled 716 adult persons nested in 36 randomly selected neighborhoods across four strata defined on density and street-connectivity – a matched sampling design. Outcome measures include two types of self-reported walking (from surveys and diaries) and so-called objective 7-day accelerometry measures. While crude differences are evident across all outcomes, adjusted effects show increased odds of travel walking in higher-density areas and increased odds of leisure walking in low-connectivity areas, but neither density nor street connectivity are meaningfully related to overall mean miles walked per day or increased total physical activity. Contrary to prior research, the authors conclude that the effects of density and block size on total walking and physical activity are modest to non-existent, if not contrapositive to hypotheses. Divergent findings are attributed to this study's sampling design, which tends to mitigate residual confounding by socioeconomic status.

## Background

While both energy intake (i.e., food consumption) and energy expenditure (i.e., physical activity) are implicated in the current obesity epidemic, US national surveillance data regarding changes in individual-level energy intake and expenditure do not appear sufficient to explain or prevent obesity [1]. Accordingly, it is worth considering more "upstream" causes, factors above and beyond an individual.

There is a strong conceptual and practical case for investigations into walking and "active transportation", and contexts in which they occur [2,3]. Despite evidence of modest increases in the prevalence of walking for physical activity, walking for transportation has shown a marked decrease in recent years. US Census data indicate that in 1970 77.7% of the working population commuted via car, truck, or van, compared with 86.5% in 1990 and 87.9% in 2000. At the same time, a decreasing proportion of the population of major metropolitan areas is using public transportation, biking, or walking to work as there has been an overall shift toward use of private cars [4,5]. A similar decrease in walking for transportation has been reported in Britain [6].

A decrease in walking for transportation may relate to an increase in the proportion of the population residing in the suburbs as well as decreasing population densities in a large number of US metropolitan areas [7]. Census data indicate that the share of housing units within metropolitan areas but outside center cities – the best approximation for measuring suburbs – increased from 19% in 1940 to 44% in 1990 and 61% in 2000. This could influence walking behaviors because suburbs are commonly less pedestrian friendly than more dense center city areas. Zoning policies in suburbs have, in some cases, resulted in little diversity of land use within a walkable distance. And suburban residential developments, particularly those built since the 1970s, tend to be separated from central business and commercial districts by major arterials and distances, such that it may be impossible or unsafe to travel to those destinations by foot or bicycle [8].

It is hypothesized that environmental design makes a difference for physical activity, particularly when it comes to integrating walking into daily life [9-11]. While most agree that social and economic variables are major factors in decisions to walk, design features are said to allow people who want to walk to do so more easily or conversely can prevent those with a marginal inclination to walk from doing so [12-14]. In fact, *New Urbanists*, proponents of transit oriented development, and others interested in sustainability and smart growth, suggest that it is time to re-develop built environments that discourage active transport and carefully develop new areas to allow them to be more pedestrian friendly [15]. They hypothesize that changes in the built environment will translate into greater active transport by individuals living and/or working in these pedestrian friendly built environments. Importantly, this line of inquiry is consistent with a growing body of epidemiologic research addressing how social and physical contexts affect health outcomes and their mediators [16].

What are the key built environment factors thought to affect walking behavior? Research in transportation and physical activity has identified: (1) density, (2) street pattern or connectivity, (3) mixed land uses or the presence of destinations, and (4) pedestrian infrastructure and design related to the issues of comfort, safety, and interest. Of these, density and street pattern are considered very important. Density is thought to be important because higher densities tend to create a critical mass of people – more people to walk, to see others walking, to feel safer. Traffic congestion also increases with population and employment density so that at a certain threshold it is more convenient to walk [17] For example, Frank and Pivo,[18] using the Puget Sound Travel Survey and census data, found more walking for shopping trips in areas with 13 or more people per acre at the census tract level. Street pattern or connectivity is thought to matter because it affects the directness of travel, making travel more or less efficient, and the number of alternative routes with implications for interest and safety [14].

At issue is the appropriate analytic methods for identifying the hypothesized multilevel effects in observational designs. In the past 6–7 years there has been a flurry of research that relies on regression adjustment, vis-à-vis the multilevel model. Oakes criticized this approach to this particular problem due to its unsupported interpolation thru design-space (ie, off-support inference due to structural confounding) [19-21]. Alternative methods, such as propensity score matching and instrumental variables analyses have been advanced and hold promise. But to date no research has addressed the identification problem from the perspective of sample designs.

This paper reports the principal outcomes from the Twin Cities Walking Study (TCWS), an investigation that relies on matched sampling to test basic hypotheses about the relationship between density, street connectivity, and walking behavior.

## Methods

The TCWS is a cross-sectional observational study specifically designed to examine the influences of the built environment on walking and physical activity. All research activities were reviewed and approved by the University of Minnesota's IRB.

### Sample Design

Sampling is especially important in observational studies on the effects of the built environment. Researchers must appreciate that "like people" do not reside in diverse neighborhoods: the rich reside in one type of neighborhood, the poor another; whites in one, blacks in another; urbanites in one, farmers in another. In fact, it is such social stratification that defines neighborhoods in the first place [22-24]. It follows that a sampling design must maximize variability in contexts while minimizing variability in resident's background characteristics; exchangeability of resident-subjects is critical [19,20,25]. Such an approach elevates the strategy of restriction over model-based statistical adjustment for confounder control.

**Figure 1:**
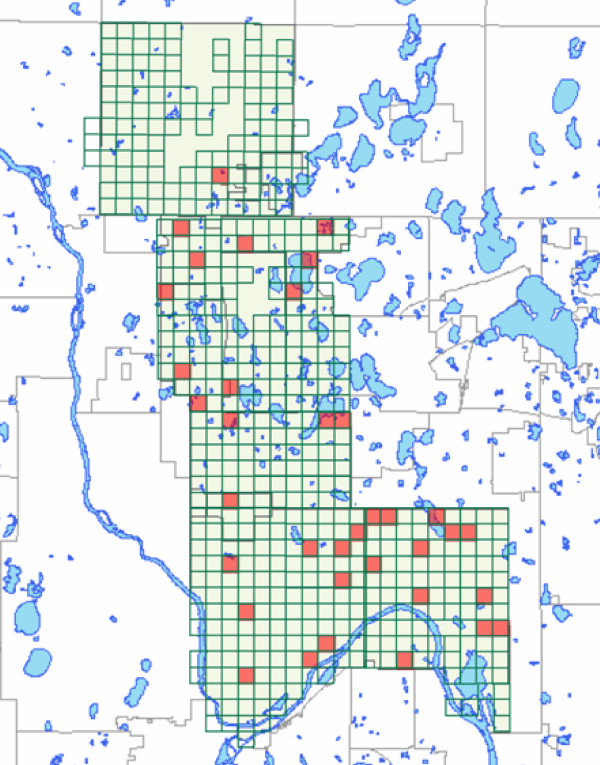
Map of Twin Cities Walking Study Neighborhood Universe (N = 130 green squares) and sample (N = 36 orange squares).

TCWS residential areas were selected from the environmentally diverse but demographically homogenous northern sector (the so-called "35W corridor") of the Minneapolis-St. Paul metropolitan area, stretching from the urban core to the urban edge, for which especially rich geographic information system (GIS) data are available (See Figure 1). Our sampling plan may be characterized as a stratified cluster design, but unlike much work in survey sampling we have direct interest in cluster (i.e., primary sampling unit) effects. One hundred thirty neighborhood areas, each 805*805 meters, were identified and stratified into high, medium or low categories across the dimensions of gross population density and street connectivity. Given disagreement on how best to do so, we operationalized street connectivity as median block size, where larger blocks reflect less connected streets, though results reported herein are robust to alternative approaches (see below). High density was defined as greater than 24.7 persons per gross hectare (ha; 1 ha is approx. 2.5 US acres) excluding water bodies only; low density was defined as less than 12.4 persons/ha. Small median block size was defined as below 2 ha, which was related to standard block sizes in the area. Large blocks were larger than 3.2 ha. These thresholds and between-strata differences are similar to those of previous researchers [10,26,27]. To maximize variability, we randomly selected M = 36 areas that ranked high or low on each of the two dimensions – we eliminated the middle strata. In the second stage, approximately 20 residents were randomly sampled from each area for a total sample size of N = 716 persons. Inclusion criteria included aged 25 year or older, primary residence in one of the 36 neighborhoods, not out of town during week of data collection, and self-reported ability to walk unaided for 20 minutes. We temporally staggered within-neighborhood subject recruitment to minimize any seasonality effects and only measured in the months April through November. Calculation of participation rates are complicated by our accepting only the first 20 volunteers per area, meaning some willing participants were turned away. However, we estimate an overall participation rate of 50%, with variability strongly correlated with the SES of area. Analyses show study subjects to be representative of their home areas (see Table 1).

**Table 1:** Comparison of final TCWS sample characteristics and 2000 U.S. Census Data

	***All Subjects***		
	**Sample**	**Census**	**Ratio**
	
**% Female**	64.81	50.94	1.27
**% aged 25–34**	19.63	23.67	0.83
**% aged 35–44**	27.31	26.40	1.03
**% aged 45–54**	24.47	21.19	1.15
**% aged 55–64**	16.22	12.41	1.31
**% aged 65–74**	8.82	8.69	1.01
**% aged 75+**	3.56	7.63	0.47
**% Caucasian**	81.21	76.26	1.06
% College*	28.90	30.59	0.94
% Married*	58.87	51.84	1.14
HH Income, $1 k*	47.41	50.01	0.95
% Own Home*	75.28	66.30	1.14
			
**Mean Ratio**			1.03
**Max Ratio**			1.31
**Min Ratio**			0.47

* Comparisons with block-group level data

### Outcome Measures

Experts in physical activity measurement have yet to determine the best ways to assess physical activity outside the domain of structured exercise sessions [28]. We employed multiple methods (2 types of self-report and accelerometry) for assessment of walking and total physical activity since each has weaknesses that can be addressed by others. For example, survey-based self-report alone could be biased due to poor recall or if the participants report what they believe the researchers wish to hear. While the objective accelerometry avoids such errors, it records all activity without regard to type or domain and does not offer reasons for activity.

The outcome measures for this analysis are limited to (1) travel walking, (2) leisure walking, (3) mean miles walked per day, and (4) total physical activity per day. Travel walking and leisure walking are self-report measures captured by the psychometrically established International Physical Activity Questionnaire – Long Form (IPAQ-LF) [29]. The measure yields quantities on the conventional metabolic equivalent times (METs) scale. METs may be understood to be the energy (oxygen) used by the body during activity per some unit of time; one MET is equivalent to the energy expended or oxygen consumed sitting in a chair for some unit of time (e.g., VO2 of approximately 3.5 mg·kg^-1^·min^-1^). Mean miles walked per day was calculated from 7-day travel and walking diaries each participant completed. Our diaries were modified versions of the National Household Travel Survey travel diary. Finally, total physical activity was measured by accelerometry. Total activity is among the most important outcome measures since it is related to many health risks and outcomes [10]. Participants wore the MTI Actigraph accelerometer (MTI Inc, Fort Walton Beach, FL) for seven consecutive days. This device records time of day and per-minute accelerations and decelerations as "activity counts". The Actigraph has been shown to be reliable in children and adolescents (ICC = 0.76 for 7 days in grades 10 to 12) [30] as well as in adults [31], when worn for 7 days. Findings also support the validity of the Actigraph in adults (r = 0.88 against treadmill walking) [32]. Yet accelerometry data are especially challenging in community studies because analysts have no information about actual use. For example, one cannot differentiate resting periods from times when a subject may have removed the device from their body. Additionally, analysts cannot differentiate activity counts related to, say, leisure walking from those related to jogging, bicycling, or any other (in)activity.

Following best-practice insights, our accelerometer data were processed such that a "valid day" was any day with more than two hours of recorded movement above an analyst defined threshold of 3 activity counts: mean total activity counts per 24-hour day were calculated by summing counts within all valid days and then dividing by the number of valid days [33]. Alternative coding (i.e., valid day definitions, activity thresholds, alternative statistics (e.g., medians, totals)) did not alter our substantive conclusions. (Programs/code are available in Stata format from the lead author by request.)

### Exposure Measures

Two environmental-level measures are central to this paper: density and street connectivity. Though there is debate regarding the best way to operationalize the construct, density is simply a number of items per unit land area. In this study, density is operationalized as total persons in housing units, as measured in the 2000 US Census at the block level per unit land area. Since the advent of widespread use of GIS in the 1990s, street pattern has been increasingly used as a measure of walkability. Recent work by Dill [34] and Steiner *et al*. [35] list numerous measures including block size or shape, intersection density or character, route directness, and access into the study area. In this paper we operationalize street connectivity as the median block size of an area, although in a related post-hoc analysis we tested eleven different measures of street connectivity including block sizes, intersection densities, and intersection types at various buffer sizes, with results similar to those presented here [36].

### Other (covariate) Measures

We collected a vast array of subject- and area-level measures for use as covariates and secondary analyses. Included are age, self-reported overall health, race/ethnicity, educational attainment, and household income. Body mass index (BMI) was objectively measured during an in-person interview.

### Analytic Procedures & Models

Analytic results include descriptive statistics and odds-ratios (OR) from ordinal logistic regression models, sometimes called proportional odds models [37]. Ordinal logistic regression was used in order to minimize the impact of measurement error in our otherwise continuous outcome measures. Given the measurement process, interval scale comparisons were not thought to be sufficiently precise. Outcome measures were classified into five ordered categories based on percentiles, but alternative classification schemes (3 to 6 percentile categories or equivalent cell frequencies) did not alter conclusions. The proportional odds assumption was met in all presented models. Conventional (asymptotic) standard error estimators are inappropriate here due to clustering of subjects within areas and, presumably, model misspecification. Accordingly, all models presented employ robust standard errors, [38] wherein study areas are identified as clusters. Note that models fit with cluster and bias-corrected bootstrapped standard errors were consistent with the presented results [39]. Additionally, conclusions are robust to model specification: adjacent-category ordinal models and negative binomial regression models (using interval-scale outcomes) reveal the same substantive relationships; so too do reasonable changes to the number and type of covariates included, including BMI, household income, and median value of owner-occupied housing. As described below, we retained "non-significant" interaction terms because we specified these tests, and designed the study around, the presented model. But again, exclusion of terms had no appreciable affect on conclusions. Stata 9.1/Se (Stata Statistical Software. College Station, TX) was used for all analyses.

## Results

Basic information about our sample is presented in Table 2. Additionally, survey data reveals an overall mean household income of $47, 410, with corresponding means for High-Density/Large-Block = $43.42 k, High-Density/Small-block = $42.55 k, Low-Density/Large-Block = $60.66 k, and Low-Density/Small-block = $52.59 k. Along with other results reported elsewhere, data show a high-degree of potential exchangeability across study areas on key mobility measures.

**Table 2:** Sample characteristics

**Measure**	**N**	**Percent**	**Min**	**Max**	**Mean**	**Median**	**Sd**
**Covariates**							
Males	702	35.19					
White persons	713	81.21					
College Degree	706	28.90					
Married	705	58.87					
Own home	704	75.28					
							
Age in years	703		24.00	86.00	47.04	45.00	13.73
Household income, $1000	557		5.00	90.00	47.41	45.00	24.76
Housing tenure in years	703		0.08	59.00	12.31	8.00	12.75
Overall health (5 = Excellent)	705		1.00	5.00	3.66	4.00	0.91
BMI	693		16.23	66.20	28.36	27.18	6.60
							
**Outcomes**							
Travel walking (mets)	702		0.00	4158.00	263.10	16.50	583.28
Leisure walking (mets)	702		0.00	4158.00	322.17	148.50	496.97
Mean miles walked per day	713		0.00	19.09	1.06	0.40	1.93
Mean total activity count per day	713		24.62	888.72	223.87	206.27	100.20
							
**Exposures**							
Density (persons/hectare)	36		3.55	48.91	21.72	22.30	12.39
Block size (hectares)	36		1.01	10.21	3.07	2.64	1.96

Table 3 presents pairwise correlations between commonly adopted outcome measures. Again, owing to concerns with measurement error, both Pearson and Spearman (rank) statistics are reported. The principal finding is that the (unconditional) pairwise correlations between outcome measures are remarkably low. As, say, travel walking increases, total physical activity need not increase. Measurement error notwithstanding, the four measures seem to tap different latent constructs, making their simultaneous use especially important to studies of this nature.

**Table 3:** Pairwise correlations between person-level outcome measures, N = 694

	**Travel Walking**	**Leisure Walking**	**Miles Walked**	**Mean Activity**
	
**Pearson correlations**				
Travel walking (mets)	1.000			
Leisure walking (mets)	0.166	1.000		
Mean miles walked per day	0.189	0.189	1.000	
Mean total activity count per day	0.077	0.264	0.305	1.000
				
**Spearman correlations**				
Travel walking (mets)	1.000			
Leisure walking (mets)	0.137	1.000		
Mean miles walked per day	0.365	0.416	1.000	
Mean total activity count per day	0.108	0.250	0.346	1.000

Table 4 presents unadjusted outcome measure means and medians across our four strata: high-density, large block (HDLB); high-density, small block (HDSB); low-density, large block (LDLB); and low-density, small block (LDSB) areas. These results are important since they clearly show point-estimate differences in both travel and leisure walking across the four strata, in ways one might anticipate. There is more travel walking in high density areas and more leisure walking in low density areas yet the table also reveals a striking lack of appreciable difference in the mean miles walked or total movement measures. Estimates from sophisticated models and/or indicators of statistical significance tend to mask the import of this simple, if not naïve, finding.

**Table 4:** Crude outcomes by density and block size strata, N = 702

	**HDLB**	**HDSB**	**LDLB**	**LDSB**
	
**Median**				
Travel walking (mets)	33.00	99.00	0.00	0.00
Leisure walking (mets)	108.90	99.00	264.00	148.50
Mean miles walked per day	0.29	0.48	0.43	0.46
Mean total activity count per day	466.80	448.50	476.50	460.07
				
**Mean**				
Travel walking (mets)	316.87	346.76	155.08	232.42
Leisure walking (mets)	252.47	274.37	393.04	370.97
Mean miles walked per day	0.88	1.30	1.02	1.05
Mean total activity count per day	466.56	461.54	476.47	459.28

HD = High density area; LD = Low density area

LB = Large block area; SB = Small block area

Adjusted odds ratios from *a priori *defined regression models are presented in Table 5 – included confounders are respondent's age, sex, race, educational attainment, marital status, home ownership status, length of tenure and overall health. Results indicate that high density areas have twice the odds of increased travel walking as low density areas, but block size has no similar effect, unless one discounts the interaction term's imprecision. Density seems to have no discernable impact on leisure walking, but larger blocks seem to increase odds ratios for leisure walking by about 40%. Neither density nor block size appears to be associated with total walking. While large block sizes do seem to increase odds ratios for residents' total physical activity by about 44% it is unclear why this is so; given other results such activity does not appear related to walking. In any case, this effect estimate is in the *opposite *direction to that proposed by new urbanists and others who believe dense highly-connected built environments promote physical activity. Overall, Table 5 suggest that while they are individually related to travel and leisure walking, respectively, neither density nor block size play a pivotal role in the total walking and only block size appears to influence total physical activity, but it is contrapositive to theoretical expectations.

**Table 5:** Odds ratios from ordinal logistic regression models

	**Travel Walking**				**Leisure Walking**			
			95% CI				95% CI	
	OR	SE	Lower	Upper	OR	SE	Lower	Upper
**High density**	1.992	0.436	1.296	3.060	0.896	0.135	0.667	1.204
**Large block-size**	0.948	0.221	0.600	1.497	1.403	0.270	0.962	2.046
**Interaction**	0.630	0.179	0.361	1.100	0.673	0.188	0.389	1.162
								
**N**	687				687			
**BIC**	1944				2172			
								
	**Total Walking**				**Total Movement**			
			95% CI				95% CI	
	OR	SE	Lower	Upper	OR	SE	Lower	Upper

**High density**	1.363	0.294	0.893	2.080	1.162	0.172	0.870	1.554
**Large block-size**	1.099	0.231	0.728	1.659	1.436	0.242	1.032	1.996
**Interaction**	0.634	0.195	0.347	1.159	0.715	0.208	0.404	1.264
								
**N**	688				689			
**BIC**	2261				2227			

notes: All models employ robust standard errors and account for clustering by focus area.

Adjusted for a subject's age, sex, race, college-degree, marital status, home ownership, home tenure, and overall health.

'Travel walking' and 'Leisure walking' are on mets scale; 'Total walking' is measured in miles; Total movement is 'mean total activity count per day' as per accelerometer

Our study was not powered for subgroup analysis but results (not shown) seem hypothesis generating. Regression models akin to those described above show high density areas are marginally associated with an increase in total walking and, in some cases, total physical activity for racial minorities, those without a college degree, the less healthy (by self-report), and the obese. In short, members of these subgroups appear to benefit from high-density areas, at least with respect to walking and physical activity.

## Conclusion

A growing body of research suggests that various aspects of the built environment effect walking, physical activity, and health, especially BMI [10,11,13,14]. At one level, such conclusions are obvious: exposure to impoverished and otherwise poor residential conditions has been known to inhibit health since Hippocrates, at least [40]. At another level, the identification of the effects of specific characteristics, such as density, neighborhood income, or even the presence of a toxic dump on human health has proved remarkably difficult, even when experimental designs have been used [19,20].

This paper reports results of an observational study specifically designed to test associations between residential density, street-connectivity and walking behavior as measured through self-report and "objective" accelerometry. Although unadjusted differences in travel, leisure and total walking, and total physical activity, are evident, regression adjusted effects suggest dense areas promote travel walking while large-block (eg, less connected) areas promote leisure walking. But the two effects appear to counterbalance one another such that total walking and total physical activity is not affected, as theory would suggest.

Why do our results contradict results of even the most recent previous studies [10,11,41]? Potential reasons include unique aspects (e.g., culture, geography, or climate) of our study region, the range of density and/or street connectivity in our target areas (i.e., not dense enough), measurement error, model misspecification, or perhaps even the particular realization of our sample. As to these explanations, our study areas are more dense than other places (eg, New Haven, CT, Worchester, MA, Ann Arbor, MI) and our areas vary widely by density as shown in Table 2. With respect to measurement error and misspecification, we took great care to employ established tools (eg, IPAQ, accelerometers) and designed this study for purposes at hand, planning all analyses in advance of data collection. Of course, it is also possible that we did not sufficiently control for all (positive) confounders, though our controls are similar to those used by others. Instead, we speculate that the central reason for the divergence is related to our research/sampling design which aimed to maximize variation in environmental attributes while minimizing the potential for confounding by background differences of residents, especially with respect to SES.

Selection bias and other issues related to SES have clouded research in the subdiscipline.[20] A few have overlooked the problem while others have attempted to use regression methods to adjust out the effects. But given the subtle and poorly understood aspects of SES, [42] the lack of a credible measure of it, and the abundantly clear structuring of environmental exposure across SES strata, we had little confidence in our ability to do this; if nothing else, the threat of residual confounding appears severe.[43] Accordingly, we identified an area of the Twin Cities region that appears relatively homogeneous with respect to SES but heterogeneous with respect to density and street connectivity. Anecdotally, our local knowledge suggests that it would be possible to for persons residing in one of our neighborhoods to reside in any other without dramatically altering their financial, social or personal characteristics – an intuitive if not practical definition of exchangeability [21]. The upshot is that studies comparing vastly different areas may suffer residual confounding by SES and non-exchangeable subjects [44,45]. Studies that maximize environmental difference while minimizing subject dissimilarity would seem to better mimic the idealized experimental trial, and therefore yield more credible effect estimates, at least estimates independent of well-known SES effects.

## Competing interests

The author(s) declare that they have no competing interests.

## References

[B1] Harnack LJ, Schmitz KH, Crawford D, Jeffery R (2006). The Role of Nutrition and Physical Activity in the Obesity Epidemic. Obesity Prevention in the 21st Century: Public Health Approaches to Tackle the Obesity Pandemic.

[B2] Owen N, Humpel N, Leslie E, Bauman A, Sallis JF (2004). Understanding environmental influences on walking; Review and research agenda. Am J Prev Med.

[B3] Pikora T, Giles-Corti B, Bull F, Jamrozik K, Donovan R (2003). Developing a framework for assessment of the environmental determinants of walking and cycling. Soc Sci Med.

[B4] McGuckin N, Srinivasan N (2003). Journey to Work Trends in the United States and its MAjor Metropolitan Areas 1960 – 2000. Report FHWA-EP-03-058.

[B5] Simpson ME, Serdula M, Galuska DA, Gillespie R, Macera C, Mack K (2003). Walking trends among U.S. adults: the Behavioral Risk Factor Surveillance System, 1987–2000. Am J Prev Med.

[B6] Lumsdon L, Mitchell J (1999). Walking, transport and health: Do we have the right prescription?. Health Promotion International.

[B7] Pendall R, Fulton W, Harrison A (2000). Losing ground on sprawl? Density trends in metropolitan America. Fair Growth Conference.

[B8] Berrigan D, Troiano R (2003). The Association Between Urban Form and Physical Activity in US Adults. American Journal of Preventive Medicine.

[B9] Frank L, Engelke P (2001). The built environment and human activity patterns: exploring the impacts of urban form on public health. Journal of Planning Literature.

[B10] Frank LD, Schmid TL, Sallis JF, Chapman J, Saelens BE (2005). Linking objectively measured physical activity with objectively measured urban form: findings from SMARTRAQ. Am J Prev Med.

[B11] Lee C, Moudon AV (2006). Correlates of Walking for Transportation or Recreation Purposes. Journal of Physical Activty and Health.

[B12] Saelens BE, Sallis JF, Black JB, Chen D (2003). Neighborhood-based differences in physical activity: an environment scale evaluation. Am J Public Health.

[B13] Sallis JF, Bauman A, Pratt M (1998). Environmental and policy interventions to promote physical activity. Am J Prev Med.

[B14] Saelens BE, Sallis JF, Frank LD (2003). Environmental correlates of walking and cycling: findings from the transportation, urban design, and planning literatures. Ann Behav Med.

[B15] Beatley T (2000). Green urbanism: Learning from European cities.

[B16] Takano T, Nakamura K, Watanabe M (2002). Urban residential environments and senior citizens' longevity in megacity areas: the importance of walkable green spaces. J Epidemiol Community Health.

[B17] Forsyth A, Oakes JM, Schmitz KH Does residential density increase walking and other physical activity?. Urban Studies.

[B18] Frank L, Pivo G (1994). Impacts of mixed use and density utilization of three modes of travel: single-occupant vehicle, transit, and walking. Transportation Research Record.

[B19] Oakes JM (2004). The (mis)estimation of neighborhood effects: causal inference for a practicable social epidemiology. Soc Sci Med.

[B20] Oakes JM (2006). Advancing Neighborhood Effects Research: Selection, Inferential Support, and Structural Confounding. International Journal of Epidemiology.

[B21] Oakes JM, Johnson PJ, Oakes JM, Kaufman JS (2006). Propensity score matching methods for social epidemiology. Methods in Social Epidemiology.

[B22] Tiebout CM (1956). A Pure Theory of Local Expenditures. The Journal of Political Economy.

[B23] Leventhal T, Brooks-Gunn J (2003). Moving to opportunity: an experimental study of neighborhood effects on mental health. Am J Public Health.

[B24] Molotch H (1976). The City as a Growth Machine: Toward a Political Economy of Place. American Journal of Sociology.

[B25] Oreopoulos P (2003). The Long-run consequences of living in a poor neighborhood. The quarterly journal of economics.

[B26] Cervero R, Duncan M (2003). Walking, bicycling, and urban landscapes: evidence from the San Francisco Bay Area. Am J Public Health.

[B27] Ewing R, Schmid T, Killingsworth R (2003). Relationship between urban sprawl and physical activity, obesity, and morbidity. Am J Health Promot.

[B28] Schutz Y, Weinsier RL, Hunter GR (2001). Assessment of free-living physical activity in humans: an overview of currently available and proposed new measures. Obes Res.

[B29] Craig CL, Marshall AL, Sjostrom M, Booth ML, Ainsworth BE, Pratt M, Ekelund U, Yngve A, Sallis JF, Oja P (2003). International physical activity questionnaire: 12-country reliability and validity. Med Sci Sports Exerc.

[B30] Trost SG, Pate RR, Freedson PS, Sallis JF, Taylor WC (2000). Using objective physical activity measures with youth: how many days of monitoring are needed?. Med Sci Sports Exerc.

[B31] Matthews CE, Ainsworth BE, Thompson RW, Bassett DR (2002). Sources of variance in daily physical activity levels as measured by an accelerometer. Med Sci Sports Exerc.

[B32] Freedson P, Malanson E, Sirard J (1998). Calibration of the Computer Science and Applications, Inc accelerometer. Med Sci Sports Exerc.

[B33] Ward DS, Evenson KR, Vaughn A, Rodgers AB, Troiano RP (2005). Accelerometer use in physical activity: best practices and research recommendations. Med Sci Sports Exerc.

[B34] Dill J (2003). Measuring network connectivity for bicycling and walking. Joint Congress of ACSP-AESOP.

[B35] Steiner R, Bond A, Miller D, Sand P (2004). Future Directions for Multimodal Areawide Level of Service Handbook: Research and Development. The Florida Department of Transportation, Office of Systems Planning, Contract BC-345-78.

[B36] Forsyth A, Schmitz K, Hearst MO, Oakes JM Design and Destinations: Factors Influencing Walking and Total Physical Activity. Urban Studies.

[B37] McCullagh P (1980). Regression models for ordinal data. Journal of the royal Statistical Society, Series B.

[B38] Woolridge JM (2002). Econometric Analysis of Cross Section and Panel Data.

[B39] Efron B, Tibshirani R (1993). An Introduction to the Bootstrap.

[B40] Hamlin C, Oakes JM, Kaufman JS (2006). The history of methods of social epidemiology to 1965. Methods in Social Epidemiology.

[B41] Li F, Fisher KJ, Brownson RC, Bosworth M (2005). Multilevel modelling of built environment characteristics related to neighbourhood walking activity in older adults. J Epidemiol Community Health.

[B42] Galobardes B, Shaw M, Lawlor D, Davey Smith G, Lynch J, Oakes JM, Kaufman JS (2006). Indicators of Socioeconomic Position. Methods in Social Epidemiology.

[B43] Kaufman JS, Cooper RS, McGee DL (1997). Socioeconomic status and health in blacks and whites: the problem of residual confounding and the resiliency of race. Epidemiology.

[B44] Greenland S, Robins JM (1986). Identifiability, exchangeability, and epidemiological confounding. Int J Epidemiol.

[B45] Rosenbaum PR (2002). Observational Studies.

